# The Radiological Evaluation of the Mastoid Process and Its Implications for Surgical Approaches

**DOI:** 10.7759/cureus.16101

**Published:** 2021-07-02

**Authors:** Ayşenur İnceoğlu, İlhan Bahşi, Mustafa Orhan, Eda Didem Yalçın, Feyza İnceoğlu

**Affiliations:** 1 Department of Anatomy, Gaziantep University, Gaziantep, TUR; 2 Dentomaxillofacial Radiology, Dokuz Eylül University, İzmir, TUR; 3 Biostatistics, Malatya Turguy Özal University, Malatya, TUR

**Keywords:** mastoid process, cone-beam computed tomography (cbct), craniofacial morphometry, posterior fossa, skull base

## Abstract

Background and objective

In various surgical procedures, in approaching the posterior fossa and the posterolateral cranial base, surface markings are used to locate the groove for transverse and sigmoid sinuses, and their junction. Determining these surface landmarks, especially the mastoid bone and its surrounding anatomical formations, is extremely valuable. The purpose of this study was to examine the anatomical features and the relationship between the mastoid process and surrounding structures on cone-beam CT (CBCT) images.

Methods

Before starting this retrospective study, approval was obtained from the Ethics Committee of Gaziantep University (approval date: 04/12/2019; number: 470). Individuals who consulted the Department of Oral and Maxillofacial Radiology of Gaziantep University's Faculty of Dentistry between 2015-2018 for any reason and whose CBCT images were taken were included in this study. Frankfort horizontal plane was used for the standardization of the images. Measurements were made on three different sections: coronal, transverse, and sagittal.

Results

The cohort consisted of 149 females and 98 males; the mean age of the participants was 45.72 ± 17.12 years. It was determined that homogeneity was achieved in terms of data distribution between genders according to age (p=0.777). Additionally, it was determined that there was a statistically significant difference between the genders in all parameters except anterior inclination angle (AIA), and higher values were found in males.

Conclusion

We believe that the results obtained from this study may contribute toward decreasing the complication rate and increasing success in surgical procedures, especially with respect to approaching the posterior fossa and the posterolateral cranial base.

## Introduction

Early diagnosis of intracranial and extracranial tumors in the posterolateral skull base can be challenging. Moreover, the complex anatomical structure of the region can create significant problems in surgical procedures [1]. Key anatomical points provide surgeons with a good understanding of the anatomical features of the relevant area, which is crucial for the surgical treatment of lesions [1,2]. In various surgical procedures, in approaching the posterior fossa and the posterolateral cranial base, surface markings are used to locate the groove for transverse and sigmoid sinuses, and their junction [3]. Determining these surface landmarks, especially the mastoid bone and its surrounding anatomical formations, is of paramount value. In this region, tumors of the glomus jugulare, which may be associated with various cranial nerves, pose a surgical challenge [4]. In such cases, the anatomical features of the mastoid bone and surrounding structures have a vital role in retrolabyrinthine mastoidectomies, which can be planned as a surgical procedure [4]. In addition, it has been reported that pneumatization and the size of the mastoid bone may be associated with many diseases such as otitis media [5]. Moreover, many studies evaluate the ability to determine gender, especially from bone remains, through the anthropometric features of the mastoid bone [6,7].

In this study, we aimed to examine the anatomical features and the relationship between the mastoid process and surrounding structures on cone-beam CT (CBCT) images as they would be helpful in various surgical procedures.

## Materials and methods

**Figure 1:**
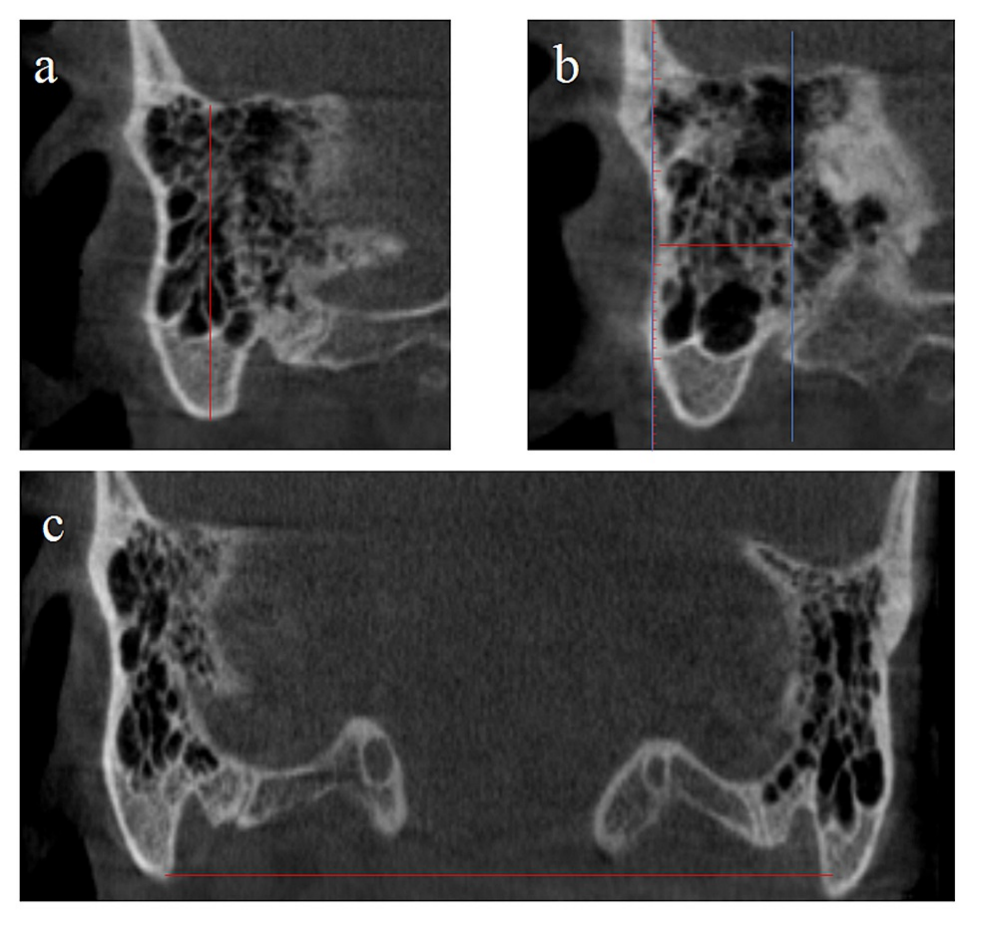
Parameters measured in coronal sections a: mastoid height; b: distance between mastoid notch and external acoustic meatus; c: bimastoid width 1

**Figure 2:**
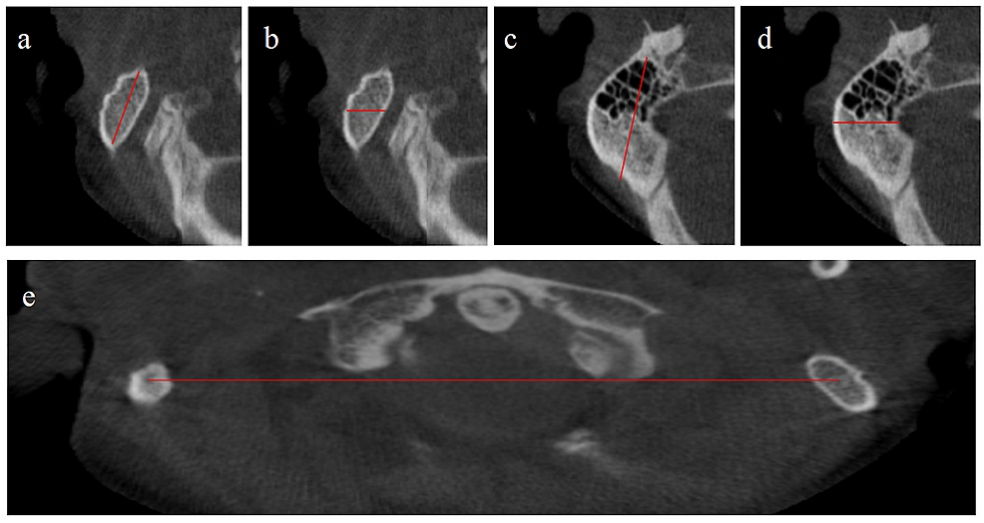
Parameters measured in transverse sections a: oblique sagittal distance; b: oblique coronal distance; c: longest oblique sagittal distance; d: longest oblique coronal distance; e: bimastoid width 2

**Figure 3:**
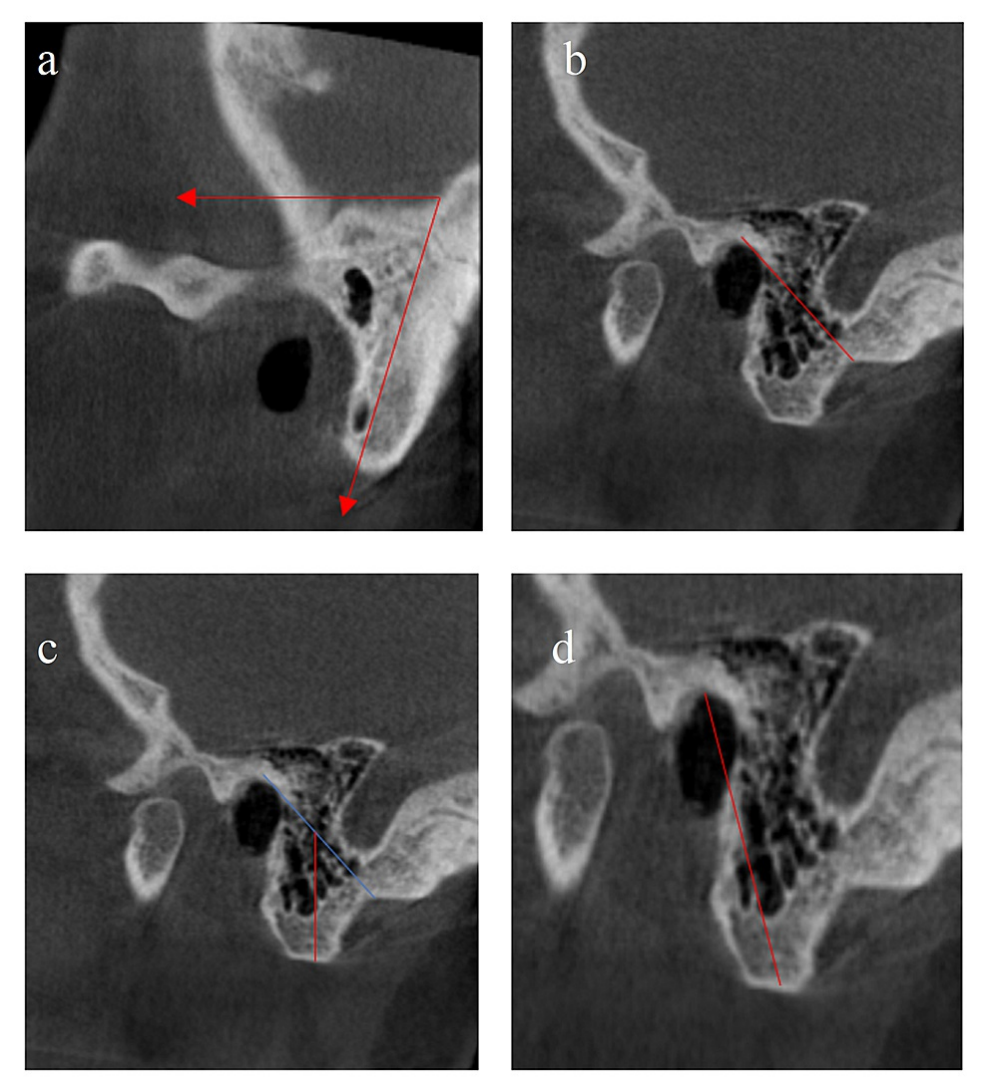
Parameters measured in sagittal sections a: anterior inclination angle; b: distance between porion and mastoid notch; c: mastoid length (blue line: porion-mastoid notch, red line: mastoid length); d: porion-mastoidale

Before starting this retrospective study, approval was obtained from the Ethics Committee of Gaziantep University (approval date: 04/12/2019; number: 470). Individuals who consulted the Department of Oral and Maxillofacial Radiology of Gaziantep University's Faculty of Dentistry between 2015-2018 for any reason and whose CBCT images were taken were included in this study. These images were evaluated by the Planmeca Romexis (Planmeca, Helsinki, Finland) program (1 mm slice thickness, 0.4 mm^3^ voxels). Any inconsistency, and unclear, incomplete, or confusing images that would hinder the measurements were not included in the study. Frankfort horizontal plane was used for the standardization of the images. Measurements were made on three different sections: coronal (Table 1 and Figure 1), transverse (Table 2 and Figure 2), and sagittal (Table 3 and Figure 3).

Parameters measured in coronal sections are presented in Table 1 and Figure 1.

**Table 1:** Parameters measured in coronal sections

Parameter	Definition
Mastoid height (MH)	Distance between mastoidale and tegmen mastoideum
Bimastoid width 1 (BW1)	Distance between right and left mastoidales
Mastoid notch-external acoustic meatus (MN-EAM)	Distance between sagittal plane passing over external acoustic meatus and mastoid notch

Parameters measured in transverse sections are shown in Table 2 and Figure 2.

**Table 2:** Parameters measured in transverse sections

Parameter	Definition
Oblique sagittal distance (OSD)	At the highest level of the mastoid notch, the longest anteroposterior length of the mastoid process
Oblique coronal distance (OCD)	At the highest level of the mastoid notch, the longest mediolateral length of the mastoid process
Longest oblique sagittal distance (LOSD)	The longest anteroposterior length of the mastoid process
Longest oblique coronal distance (LOCD)	The longest mediolateral length of the mastoid process
Bimastoid width 2 (BW2)	Distance between right and left mastoidales

Parameters measured in sagittal sections are shown in Table 3 and Figure 3.

**Table 3:** Parameters measured in sagittal sections

Parameter	Definition
Anterior inclination angle (AIA)	Angle between the Frankfort horizontal plane and the plane passing through the mastoidale
Porion-mastoid notch (Po-MN)	Distance between the porion and the most lateral point of the mastoid notch
Mastoid length (ML)	Distance between the midpoint of porion-mastoid notch and mastoidale
Porion-mastoidale (Po-M)	Distance between porion and mastoidale

Statistical analysis

All statistical analyses were performed using the SPSS Statistics software version 25.0 (IBM, Armonk, NY). The significance level (p-value) was taken as 0.05 for the comparison tests. The conformity of the data to the normal distribution was checked with the Kolmogorov-Smirnov test. Comparisons were done between independent pairs; since the assumption of normality was provided, comparisons between groups were made with the significance test (t-test) with regard to the difference between the two means.

## Results

Our cohort comprised 149 females and 98 males. The mean age of the participants was 45.72 ± 17.12 years. It was determined that homogeneity was achieved in terms of data distribution between genders according to age (p=0.777). The results of the parameters measured in coronal sections are given in Table 4, those in transverse sections in Table 5, and those in sagittal sections in Table 6. It was determined that there was a statistically significant difference between the genders in all parameters except anterior inclination angle (AIA), and higher values were found among males.

**Table 4:** Results of parameters measured in coronal sections *Statistically significant F: female; M: male; R: right, L: left; MH: mastoid height; BW1: bimastoid width 1; MN-EAM: distance between mastoid notch and external acoustic meatus; SD: standard deviation

Parameter		Group	N	Mean ± SD	P-value
MH (mm)	R	F	149	35.64 ± 4.21	0.001*
		M	98	40.78 ± 4.59	
	L	F	149	36.25 ± 4.75	0.001*
		M	98	41.14 ± 4.70	
BW1 (mm)		F	149	102.03 ± 3.75	0.001*
		M	98	105.86 ± 4.40	
MN-EAM (mm)	R	F	149	14.23 ± 2.34	0.001*
		M	98	15.25 ± 2.23	
	L	F	149	13.76 ± 2.05	0.001*
		M	98	14.65 ± 1.98	

**Table 5:** Results of parameters measured in transverse sections *Statistically significant F: female; M: male; R: right; L: left; OSD: oblique sagittal distance; OCD: oblique coronal distance; LOSD: longest oblique sagittal distance; LOCD: longest oblique coronal distance; BW2: bimastoid width 2; SD: standard deviation

Parameter		Group	N	Mean ± SD	P-value
OSD (mm)	R	F	149	17.66 ± 3.15	0.001*
		M	98	20.84 ± 3.69	
	L	F	122	16.76 ± 2.91	0.001*
		M	49	18.74 ± 3.29	
OCD (mm)	R	F	149	10.18 ± 1.93	0.001*
		M	98	11.70 ± 2.06	
	L	F	122	9.95 ± 1.71	0.001*
		M	49	11.21 ± 2.18	
LOSD (mm)	R	F	149	29.24 ± 4.38	0.001*
		M	98	30.99 ± 3.88	
	L	F	79	27.38 ± 4.02	0.046*
		M	31	29.00 ± 2.99	
LOCD (mm)	R	F	148	17.47 ± 3.66	0.001*
		M	98	19.51 ± 4.14	
	L	F	79	16.15 ± 3.24	0.010*
		M	31	17.86 ± 2.60	
BW2 (mm)		F	149	102.46 ± 3.70	0.001*
		M	97	106.14 ± 4.35	

**Table 6:** Results of parameters measured in sagittal sections *Statistically significant F: female; M: male; R: right; L: left; AIA: anterior inclination angle; Po-MN: distance between porion and mastoid notch; ML: mastoid length; Po-M: porion-mastoidale; SD: standard deviation

Parameter		Group	N	Mean ± SD	P-value
AIA (°)	R	F	148	69.86 ± 2.69	0.822
		M	98	69.78 ± 2.94	
	L	F	149	70.29 ± 2.77	0.711
		M	98	70.15 ± 2.68	
Po-MN (mm)	R	F	149	27.56 ± 3.30	0.001*
		M	98	30.35 ± 3.99	
	L	F	149	27.23 ± 3.55	0.001*
		M	98	29.58 ± 3.72	
ML (mm)	R	F	149	17.22 ± 2.63	0.001*
		M	98	20.33 ± 2.91	
	L	F	149	17.40 ± 2.61	0.001*
		M	98	20.63 ± 3.0	
Po-M (mm)	R	F	149	28.22 ± 3.46	0.001*
		M	98	32.37 ± 4.20	
	L	F	149	28.47 ± 3.85	0.001*
		M	98	32.93 ± 4.31	

## Discussion

The mastoid process is an important anatomical entity that has been extensively studied in the literature. It is a point of common interest for anatomists, otolaryngologists, radiologists, neurosurgeons, anthropologists, and forensic experts. The important structures in its surroundings, the muscles it makes origo and insertio, and the differences in size depending on age and gender are the essential features of this structure [6-9]. Moreover, due to its durability and sheltered location, the mastoid process can be used for sex determination from bone remains in forensic medicine [7,8].

In addition, the mastoid process can be used as a surface landmark for approaching the posterior fossa and skull base in surgical procedures [3]. Because of its close relationship with some cranial nerves such as the accessory nerve and many vital structures, it is crucial to know the anatomical features of the region in surgical procedures. Variations of this region can determine the type of surgical procedure to be employed. Therefore, knowing the anatomy of this region well will decrease the complication rate and increase the success of surgical procedures.

In the literature, it is seen that the mastoid process and surrounding structures have been examined through various methods such as lateral cephalogram [10], CT [11], CBCT [12], and multidetector CT (MDCT) [9] images and direct measurement on the dry bones [8]. However, it is known that it is challenging to precisely know the features such as ethnicity, age, and gender in studies examining dry bones [13]. In addition, CBCT images are more advantageous than cephalogram studies due to the possibilities offered by three-dimensional examination and CT images as well as the lower radiation exposure [14]. Because of these advantages, CBCT images were examined in this study. Moreover, Buran et al. [15] have reported that their morphometric analysis based on imaging modalities is an opportunity to build comprehensive social databases.

In the literature, it has been reported that the mastoid process and surrounding structures are more extensive in males because the male skull is larger than that of females [15]. Consistent with this, all parameters except AIA were found to be greater in males than females in this study.

Gopal et al. [16] have reported the mastoid height as 33.06 ± 3.22 mm in males and 27.49 ± 3.53 mm in females. Bhayya et al. [17] have reported the mastoid height as 31.48 ± 4.68 mm on the right side and 30.92 ± 4.56 mm on the left side in males, and 27.60 ± 3.51 mm on the right side and 28.40 ± 4.80 mm on the left side in females. In this study, those were found to be slightly larger than what is reported in the literature: 40.78 ± 4.59 mm on the right side and 41.14 ± 4.70 mm on the left side in males, and 35.64 ± 4.21 mm on the right side and 36.25 ± 4.75 mm on the left side in females.

Buran et al. [15] have documented the bimastoid width to be 108.5 ± 4.3 mm in males and 100.8 ± 4.1 mm in females. Marinescu et al. [18] have reported this length as 105.4 mm in males and 99.6 mm in females. Gopal et al. [16] have reported this length as 103.39 ± 5.04 mm in males and 98.30 ± 4.71 mm in females. Our findings are in line with the literature, as it was found to be 105.86 ± 4.40 mm in coronal sections and 106.14 ± 4.35 mm in sagittal sections in males, and 102.03 ± 3.75 mm in coronal sections and 102.46 ± 3.70 mm in sagittal sections in females.

Saini et al. [19] have reported the porion-mastoidale to be 31.775 ± 3.076 mm in males and 27.987 ± 3.47 mm in females. Similarly, in this study, it was found to be 32.37 ± 4.20 mm on the right side and 32.93 ± 4.31 mm on the left side in males, and 28.22 ± 3.46 mm on the right side and 28.47 ± 3.85 mm on the left side in females.

Limitations

The relatively small sample size in this study can be considered a methodological limitation. In addition, since the study was retrospective in design, we were unable to obtain precise information about the ethnic origins of the individuals.

## Conclusions

In various surgical procedures, in approaching the posterior fossa and the posterolateral cranial base, surgeons should have a clear idea about the variations of the mastoid process and surrounding anatomical structures. In this way, the risk of complications during the procedure can be reduced and the success rate will increase. We think that the results obtained in this study may contribute to decreasing the complication rate and increasing success, especially in approaching the posterior fossa and the posterolateral cranial base. On the other hand, we think that the anatomy of the mastoid process and surrounding structures needs to be further evaluated in larger sample sizes and through more comprehensive studies among various ethnic groups.
